# Clinical profile of pulmonary aspergilloma complicating residual tubercular cavitations in Northen Indian patients

**DOI:** 10.4103/0970-2113.71947

**Published:** 2010

**Authors:** P. R. Gupta, Aruna Vyas, R. C. Meena, Shivraj Sharma, N. Khayam, I. M. Subramanian, D. Kanoongo, V. Solanki, A. Bansal

**Affiliations:** *Department of Pulmonary Medicine, SMS Medical College, Jaipur, India*; 1 *Department of Microbiology, SMS Medical College, Jaipur, India*; 2 *Department of Chest and TB, SMS Medical College, Jaipur, India*

**Keywords:** Aspergilloma, ball like lesions, recent thickening of cavity wall

## Abstract

**Background:**

Little is known regarding the clinical profile of Aspergilloma in Indian patients. Such a study was undertaken at Hospital for Chest and TB, Jaipur.

**Materials and Methods:**

Old, treated patients of pulmonary tuberculosis showing ball like lesion/s inside cavity/ies or a recent thickening of cavity wall were enrolled. Morning sputa samples were collected in the patients who were able to raise sputum and were examined by KOH mount and fungal culture. Serum anti-aspergillus antibodies were estimated in all the patients. Twenty normal healthy subjects were included to serve as control. All patients showing a positive or borderline positive serology were diagnosed as pulmonary aspergilloma (PA group). The remaining patients formed the non-aspergilloma group (Non PA group).

**Results:**

A total of 98 study patients could be classified as PA group (54 patients by serology alone, 44 patients by serology as well as sputum culture). The remaining 152 patients were classified as non PA group. Hemoptysis alone or along with other chest symptoms was significantly more common in PA group as compared to non PA group patients (*P*<0.001), more so in those with ball like lesions. But chest symptoms other than hemoptysis were more common in non PA group. Within the PA group, 21 (13 with ball like lesions and 8 with thickening of cavity wall) had clinical symptoms suggestive of CNPA and two patients (one each with ball like lesions and thickening of cavity wall) had clinical symptoms suggestive of ABPA.

**Conclusion:**

The clinical profile of pulmonary Aspergilloma in Indian patients is very protean ranging from saprophytic disease to CNPA and less commonly to ABPA.

## INTRODUCTION

Pulmonary aspergilloma is a saprophytic form of aspergillosis. It results from in growth of Aspergillus in damaged broncho-pulmonary tissues caused by various lung diseases, most commonly, the residual tubercular cavities[1] Radiologically it presents as a single or multiple ball like lesions inside a cavity/cavities, partially surrounded by a radiolucent crescent (Monod’s sign).[2] A recent thickening of the wall of a pre-existing cavity and/or pleural thickening may indicate early disease.[3–5] Many of these patients either do not expectorate or their sputa are negative for mycelia.[6,7] Diagnosis of the disease is thus mainly based on detection of serum anti-aspergillus antibodies.

In a review of 9 studies on aspergilloma, Glimp *et al*.[8] stated that hemoptysis was the most frequent symptom but cough (dry or productive), dyspnoea, malaise and weight loss might be present. Other associated symptoms included wheezing, chest pain and fever. Many patients remained asymptomatic. However, all the patients in these studies were not confirmed by serology or culture. Further, clinical studies on patients suffering from pulmonary aspergilloma are lacking in India. A study was therefore, undertaken at the Department of TB and Chest diseases, SMS Medical College, Jaipur, to know the clinical profile of these patients.

## MATERIALS AND METHODS

All old treated patients of pulmonary tuberculosis with disease duration of more than two years and showing ball like lesions inside old cavities or a recent thickening of cavity wall were enrolled and subjected to further clinical assessment and laboratory investigations including hemogram, blood sugar and urea, HIV serology, urine complete examination and sputum for Gram’s stain, AFB and pyogenic culture and sensitivity. Patients showing evidence of active pulmonary tuberculosis, AIDS or other immuno-suppressive disorder, diabetes mellitus, chronic renal or liver disease or showing clinico-radiological evidence of improvement on being administered antibiotics, were excluded from the study. The remaining patients formed the study group. Twenty normal healthy volunteers were included to serve as controls.

Morning sputa samples were collected for two consecutive days in those patients who were able to raise sputum and were examined by KOH mount and fungal culture.

Venous blood sample was drawn in all the study patients and controls. Serum was separated and diluted using diluent buffer. It was then subjected to measurement of antibody titers by ELISA technique using kits supplied by IBL Immuno Biological Laboratories, Hamburg (Germany) as per their manual. In short, 100 *µ*L each of standard and diluted sample was added into the respective wells of the micro titer plate and covered with adhesive foil. It was then incubated for 60 min at 18–25°C. The plate was then washed three times with 300 *µ*L of diluted wash buffer. Excess solution was removed by tapping the inverted plate on a paper towel. 100 *µ*L of enzyme conjugate was added into each well. The plate was again covered with new adhesive foil and incubated for 30 minutes at 18–25°C. The plate was again washed three times with 300 *µ*L of diluted Wash buffer. Excess solution was removed by tapping the inverted plate on a paper towel. 100 *µ*L of TMB substrate solution was added into each well and incubated for 20 minutes at 18–25°C in the dark without adhesive foil. Substrate reaction was stopped by adding 100 *µ*L of TMB stop solution into each well. The contents were mixed by gently shaking the plate. Optical density was measured with a photometer at 450 nm (Reference-wave length: 600–650 nm) within 60 min after adding the stop solution. The calculated absorptions for the patients’ sera were compared with the value for the cut off standard. The test was read as positive, if the absorption value of the sample was higher by 50% or more as the standard cut off but if it was lower by 20% or more, it was considered as negative. A value of 20– 50% above the cut-off value was considered as borderline positive. A value of 20% ± around the cut-off was considered as gray zone and a repeat test was done in these cases. A gray zone on repeat test was read as negative.

All patients showing a positive or borderline positive serology were diagnosed as pulmonary aspergilloma (PA group). The remaining patients formed the non-aspergilloma group (Non PA group). The clinical profiles of the two groups were tabulated and analyzed.

## RESULTS

The intake of patients for the study started in March 2004 and was completed in December 2007. After exclusions, 250 patients were left for inclusion in the study. The mean age of the study patients was 38.0±5.8 years. Males outnumbered females by a ratio of 19:6. Thirteen healthy males and seven females, with a mean age of 37.4±4.7 served as control. Hemoptysis of varying severity was the most common symptom (112 patients). Cough, pain chest, shortness of breath, sputum, weight loss and fever were the other symptoms, being present in 65, 43, 33, 23, 23 and 21 patients respectively; 68 patients had more than one symptom. Seventy eight patients were asymptomatic.

Only 88 study patients were able to raise sputum. KOH mount of their sputa showed fungal hyphae in 22 samples and pseudohyphae in 11. Five sputa samples were positive for hyphae as well as pseudohyphae. Culture of the sputum showed Aspergilli in 46, Candida in 11 and other fungi in nine. No growth was seen in 29 samples and contamination was evident in two samples. Nine patients had grown more than one fungi. Different Aspergilli included A. fumigatus in 37, A. niger in six and A. flavus in three.

Serology was strongly positive in 86, borderline positive in 12 and negative in the remaining 152 patients. It was negative in all the control subjects. Out of the total 250 patients, only 98 patients qualified as having aspergilloma (PA group: 54 patients by serology alone, 44 patients by serology as well as sputum culture). The remaining 152 patients were classified as non PA group.

The distribution of patients in PA and non PA group according to symptoms is shown in Table 1. Symptoms were significantly more common in PA group as compared to non PA group patients (*P*<0.001). This difference was mainly due to higher number of patients with hemoptysis alone or along with other chest symptoms in the PA group. Within the PA group, hemoptysis was more common in those with ball like lesions. Chest symptoms without hemoptysis were more common in non PA group.

**Table 1:** Clinical symptoms in the two groups

Symptoms	PA group		Non PA group		Total
	Ball like lesions	Thickening of cavity wall	Ball like lesions	Thickening of cavity wall
Haemoptysis alone	50	05	02	20	77
Chest symptoms and haemoptysis	19	11	02	03	35
Chest symptoms but no haemoptysis	02	09	02	47	60
Fever/Weight loss*	12	09	1	1	23
No symptoms	02	02	05	69	-
Total	72	26	12	140	252

* All these patients also had chest symptoms

Within the PA group, 21 (13 with ball like lesions and 8 with thickening of cavity wall) had clinical symptoms suggestive of chronic necrotizing pulmonary aspergillosis (CNPA)[9] namely cough, sputum and/or hemoptysis along with weight loss, fever and/or malaise and two patients (one each with ball like lesions and thickening of cavity wall) had clinical symptoms suggestive of allergic broncho-pulmonary aspergillosis (ABPA)[10] namely cough, shortness of breath, and wheezing along with peripheral eosinophillia.

## DISCUSSION

A total of 250 suspected cases of Aspergilloma were evaluated but only 98 qualified themselves as suffering from the disease, on the basis of serology (PA group). This includes 26 out of 166 patients with a recent thickening of the wall of a pre-existing cavity. This is in line with earlier studies.[3–5] But 12 patients with typical ball like lesions and 140 patients with a recent thickening of the wall of a pre-existing cavity could not be confirmed as aspergilloma. The radiological lesions in these non PA group patients were either due to organisms other than A. fumigatus or were non specific in nature. Else, it is possible that the fungi in these patients was mainly in the commensal phase and were thus unable to incite sufficient host response. Infrequent symptoms in non PA group patients as compared to PA group patients, points to the later hypothesis.

The clinical profile of the PA group patients in this study was variable. Fifty patients presented with hemoptysis alone, 23 other patients had hemoptysis and/or other chest symptoms; 21 patients had clinical symptoms suggestive of CNPA; two patients had clinical symptoms suggestive of ABPA and four patients were asymptomatic. Majority of the above manifestations in this study are in line with earlier studies on aspergilloma, which found hemoptysis as the most common symptom (50 to 80%).[8,11,12] Glimp *et al*.[8] noted that a few of their patients were wheezing and these were actually the cases of ABPA. Kohno *et al*.[12] reported that many of their patients of Aspergilloma also had clinical features, suggestive of CNPA. Thus the clinical presentation of aspergilloma is protean. It suggests that pulmonary aspergillosis is a disease with continued spectrum and its division of into allergic, saprophytic and invasive forms is indistinct. Pulmonary aspergilloma is no exception to this.

In conclusion, the clinical profile of pulmonary Aspergilloma in Indian patients is very protean ranging from saprophytic disease to CNPA and less commonly to ABPA.
